# Computed tomography assessment of exogenous surfactant-induced lung reaeration in patients with acute lung injury

**DOI:** 10.1186/cc9186

**Published:** 2010-07-15

**Authors:** Qin Lu, Mao Zhang, Cassio Girardi, Belaïd Bouhemad, Jozef Kesecioglu, Jean-Jacques Rouby

**Affiliations:** 1 Multidisciplinary Intensive Care Unit, Department of Anesthesiology and Critical Care Medicine, Assistance Publique-Hôpitaux de Paris, La Pitié-Salpêtrière Hospital, UPMC Univ Paris 06, 47-83 boulevard de l'hôpital, 75013 Paris, France; 2 Department of Emergency Medicine, Second Affiliated Hospital, Zhejiang University, School of Medicine, 88 Jiefang Road, 310009 Hangzhou, China; 3 Department of Anesthesiology, Federal University of Sao Paulo, Escola Paulista de Medicina, Rua Napoleão de Barros, 715 - 5° andar, Vila Clementino CEP 04024002 São Paulo, Brazil; 4 Department of Intensive Care Medicine, University Medical Center Utrecht, Heidelberglaan, 100, 3584 CX Utrecht, The Netherlands

## Abstract

**Introduction:**

Previous randomized trials failed to demonstrate a decrease in mortality of patients with acute lung injury treated by exogenous surfactant. The aim of this prospective randomized study was to evaluate the effects of exogenous porcine-derived surfactant on pulmonary reaeration and lung tissue in patients with acute lung injury and acute respiratory distress syndrome (ALI/ARDS).

**Methods:**

Twenty patients with ALI/ARDS were studied (10 treated by surfactant and 10 controls) in whom a spiral thoracic computed tomography scan was acquired before (baseline), 39 hours and 7 days after the first surfactant administration. In the surfactant group, 3 doses of porcine-derived lung surfactant (200 mg/kg/dose) were instilled in both lungs at 0, 12 and 36 hours. Each instillation was followed by recruitment maneuvers. Gas and tissue volumes were measured separately in poorly/nonaerated and normally aerated lung areas before and seven days after the first surfactant administration. Surfactant-induced lung reaeration was defined as an increase in gas volume in poorly/non-aerated lung areas between day seven and baseline compared to the control group.

**Results:**

At day seven, surfactant induced a significant increase in volume of gas in poorly/non-aerated lung areas (320 ± 125 ml versus 135 ± 161 ml in controls, *P *= 0.01) and a significant increase in volume of tissue in normally aerated lung areas (189 ± 179 ml versus -15 ± 105 ml in controls, *P *< 0.01). PaO_2_/FiO_2 _ratio was not different between the surfactant treated group and control group after surfactant replacement.

**Conclusions:**

Intratracheal surfactant replacement induces a significant and prolonged lung reaeration. It also induces a significant increase in lung tissue in normally aerated lung areas, whose mechanisms remain to be elucidated.

**Trial registration:**

NCT00742482.

## Introduction

Acute respiratory distress syndrome (ARDS) or acute lung injury (ALI) is characterized by hypoxemia, high permeability type pulmonary edema, decreased lung compliance and loss of aeration. Inactivation or deficiency of surfactant is directly involved in ARDS pathophysiology [1]. Pre-clinical experiments show that mechanical ventilation itself can also have a deleterious impact on endogenous surfactant [2,3].

Currently, intratracheal replacement of surfactant is recognized as the standard therapy for premature neonates and children with acute respiratory failure [4,5]. In patients with ARDS/ALI, despite the efficacy of surfactant on arterial oxygenation and lung compliance [6], randomized trials have failed to demonstrate a decrease in mortality [7,8]. Inadequate doses of surfactant and short treatment duration may account for the lack of beneficial effect on mortality rate [9,10]. Administration of natural surfactant rather than synthetic surfactant increases the treatment efficacy and decreases mortality rates in neonates [11]. A recent randomized multicenter trial, however, failed to demonstrate any improvement in mortality following the bolus administration of exogenous natural porcine surfactant in patients with early ALI/ARDS [12]. Moreover, oxygenation was not improved by surfactant replacement in this trial. In ARDS/ALI, loss of lung aeration does not have a uniform distribution. In the supine position, aeration loss largely predominates in the lower lobes as a result of external compression by the abdomen and heart [13,14]. The deficiency of surfactant also contributes to the loss of lung aeration. As a result, in a vast majority of patients fulfilling the ALI/ARDS criteria, upper lobes remain entirely or partly normally aerated [15]. During mechanical ventilation with positive end-expiratory pressure (PEEP), alveolar recruitment and lung overinflation occur simultaneously in different parts of the lung [16,17]. If natural surfactant administered by intratracheal route reaches the distal lung, it should logically reaerate nonaerated lung regions, induce a more homogenous regional distribution of tidal volume and PEEP, and consequently result in a reduction of mechanical ventilation-induced lung injury.

Computed tomography (CT) is the reference method for measuring alveolar recruitment [18] because it provides the possibility of performing a regional analysis taking into account normally and poorly or nonaerated lung regions separately. Alveolar recruitment can be defined as the volume of gas penetrating into poorly and nonaerated lung areas following various therapies such as PEEP, recruitment maneuver or surfactant administration. Based on this CT method, we undertook a prospective randomized study aimed at evaluating the effect of porcine-derived lung surfactant administered by the intratracheal route on lung reaeration in patients with ARDS/ALI.

## Materials and methods

### Study design

The present study is a part of an international, multicenter, randomized, controlled, open, parallel group study conducted between January 2003 and May 2004 [12]. Twenty mechanically ventilated critically ill patients admitted to the multidisciplinary ICU of La Pitié-Salpêtrière Hospital, University Pierre et Marie Curie, Paris, France, for ALI/ARDS were included in the study and randomized either to the surfactant group (three doses of surfactant in addition to usual care,* n* = 10) or to the control group (usual care alone,* n *= 10). Inclusion was restricted to the first 60 hours from the start of mechanical ventilation. Exclusion criteria were: age 18 years or less, acute bronchial asthma attack or suspected pulmonary thrombo-embolism, daily medication for chronic obstructive pulmonary disease at time of admission, need for mechanical ventilation for more than 48 hours continuously within one month prior to the current ventilation period, pneumonectomy or lobectomy, untreated pneumothorax, tracheostomy, surgical procedures under general anesthesia performed within six hours, mean arterial blood pressure below 50 mmHg despite adequate fluid administration and/or need for vasoactive drugs, partial pressure of arterial oxygen (PaO_2_) below 75 mmHg with a fraction of inspired oxygen (FiO_2_) of 1.0 not responding to adjustment of PEEP, head injury, life expectancy less than three months due to primary disease and treatment with any investigational drug within the previous four weeks. The institutional review board of La Pitié-Salpêtrière approved the study protocol. Two informed consents were obtained from each patient or their next of kin: one for inclusion in the international, multicenter, randomized, controlled study conducted between January 2003 and May 2004 [12] and another for the present study.

### Surfactant administration

A freeze-dried natural surfactant isolated from pig lungs (HL-10, Leo Pharmaceutical Products, Ballerup, Denmark; Halas Pharma GmbH, Oldenburg, Germany) composed of approximately 90 to 95% phospholipids, 1 to 2% surfactant hydrophobic proteins (surfactant proteins SP-B and SP-C) and other lipids was administered to the patients. The product was delivered as a solution containing 50 mg/ml of HL-10 (100 ml vials containing 3 g of HL-10 dispersed in 60 ml warm 37 to 40°C saline). Baseline was defined as the time after randomization preceding the first large bolus of surfactant. Up to three doses of HL-10, totalling a maximum cumulative amount of 600 mg/kg (200 mg/kg/dose) were instilled at 0 hour, 12 and 36 hours thereafter. Before each large bolus, patients were sedated and paralyzed. HL-10 was then placed in two 300 ml syringes, with half of the total dose in each. The mechanical ventilator was set on volume control mode with a tidal volume of 6 ml/kg predicted body weight (PBW), FiO_2 _of 1.0 and PEEP left unchanged. The patient was turned to one side, the endotracheal tube was clamped at expiratory hold, the mechanical ventilator was disconnected from the patient, and the HL-10 injected into the endotracheal tube as fast as possible. The patient was reconnected to the ventilator, the tube was unclamped and the tidal volume was temporarily increased to 12 ml/kg PBW with PEEP reduced to 5 cmH_2_O to optimize the pulmonary distribution of HL-10. After five breaths, PEEP was set 5 cmH_2_O above pre-HL-10 administration values for 30 minutes, to avoid transient hypoxemia. After all the HL-10 had disappeared from the tube, the patient was turned back to the supine position and the tidal volume was put back to 6 ml/kg PBW. After a steady state was obtained, the patient was turned to the opposite side and the administration process was repeated to the other lung.

### Computed tomography measurement of lung reaeration

Each patient was transported to the Department of Radiology by two physicians (QL, MZ, CG, BB). Spiral CT sections were acquired from the apex to the diaphragm using a spiral Tomoscan SR 7000 (Philips, Eindhoven, The Netherlands) at PEEP 10 cmH_2_O at baseline, 39 hours (H39) or within 3 hours after the third bolus of HL-10 for surfactant group and day 7. During the acquisition, airway pressure was monitored to ensure that PEEP 10 cmH_2_O was actually applied. Contiguous axial 5 mm thick sections were reconstructed from the volumetric data using standard filter in order to avoid an artifactual increase in the hyperinflated compartment [19].

#### Computed tomography measurement of lung, gas and tissue volumes

CT data were analyzed using a specifically designed software (Lungview, Institut National des Télécommunications, France) including a color-coding system [20]. The following lung compartments were identified: hyperinflated, made up voxels with CT numbers between -1000 and -900 HU; normally aerated made up voxels with CT numbers between -900 and -500 HU; poorly aerated made up voxels with CT numbers between -500 and -100 HU; nonaerated made up voxels with CT numbers between -100 and +100 HU. Using the color-coding system of Lungview, each nonaerated voxel was colored in red, each poorly aerated voxel in light gray, each normally aerated voxel in dark gray and each hyperinflated voxel in white. The overall volume of gas present in both lungs at PEEP 10 cmH_2_O was defined as end-expiratory lung volume. Volumes of gas and tissue and hyperinflated lung volume of the whole lung were measured as described in the additional file at baseline, H39 and day 7 [see Additional file 1].

#### Computed tomography measurement of surfactant-induced lung reaeration

**Figure 1:**
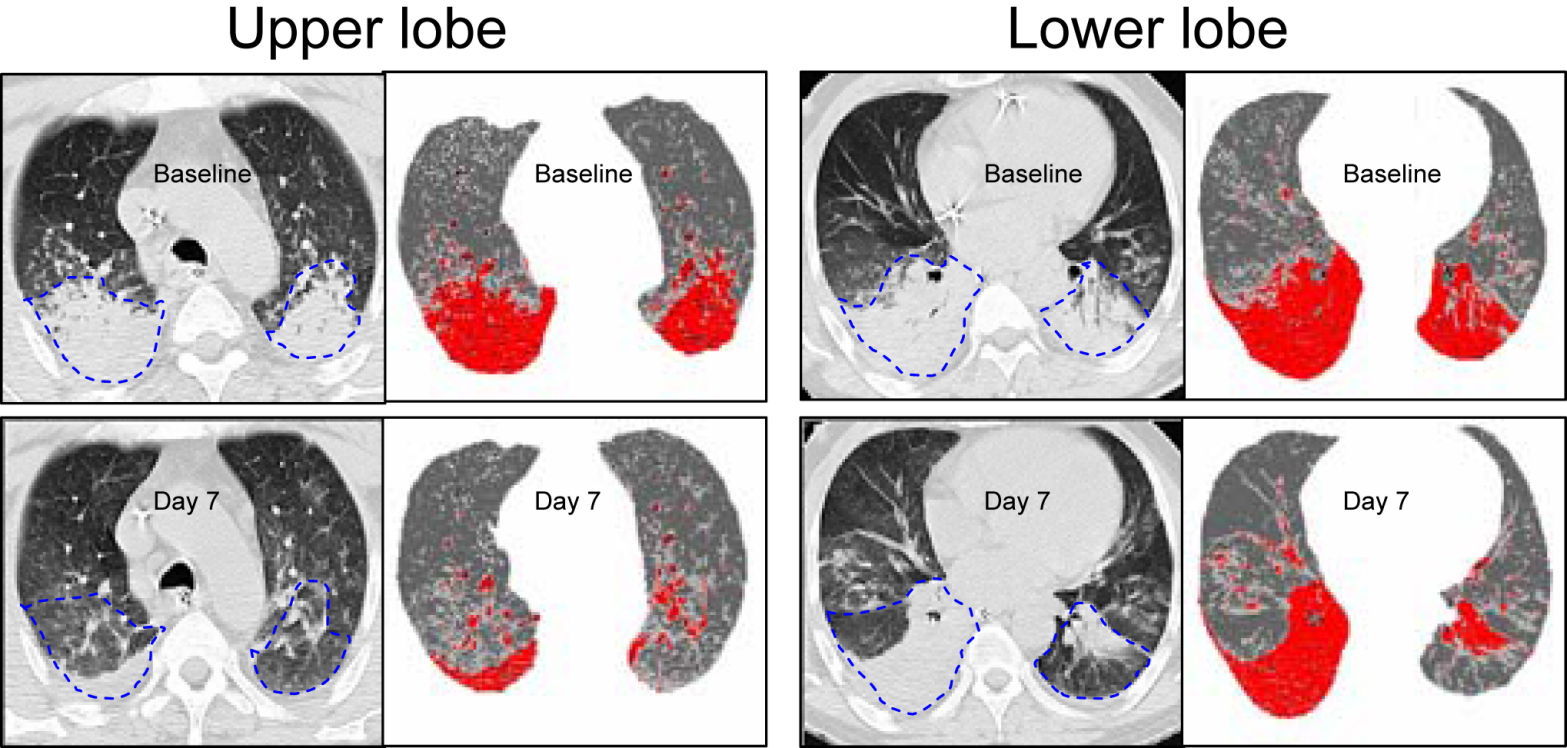
**Representative CT sections of upper and lower lobes obtained at baseline and day 7 in a patient with acute respiratory distress syndrome**. Computed tomography (CT) sections at baseline and day 7 are at the same lung region as attested by the anatomical landmarks present on the rough images at baseline and day 7 (aortic arch and vascular divisions for upper lobe CT sections and vascular divisions for the lower lobe CT sections). As previously described [18], poorly and nonaerated lung areas of right and left upper and lower lobes are manually delineated (dashed line) at baseline (before HL-10 administration) with the aid of the software Lungview) that identifies poorly and nonaerated lung areas in light gray and red, respectively. Delineation performed at baseline is manually 'transposed' to the CT section corresponding to the same anatomical level obtained at day 7. Surfactant-induced lung reaeration is defined as the increase in gas volume within the delineated zone between day 7 and baseline. The same process is repeated on each CT section in order to assess overall surfactant-induced lung reaeration.

Surfactant-induced lung reaeration was computed on all CT sections according to a method proposed by Malbouisson and colleagues for measuring PEEP-induced alveolar recruitment [18]. Such a method is based on the concept of measuring reaeration not only in nonaerated but also in poorly aerated lung regions on the whole lung. Accordingly, surfactant-induced reaeration was defined as the increase in the volume of gas entering nonaerated and poorly aerated lung regions after three doses of surfactant administration (day 7) compared with baseline. In the control group, lung reaeration was computed as the increase in gas volume within poorly and nonaerated lung regions between day 7 and baseline. The detail regional CT analysis is described in Figure 1.

#### Computed tomography assessment of lung distribution of surfactant

In both surfactant and control patients, right upper and middle lobes, right lower lobe, left upper lobe and left lower lobe were analyzed separately at baseline and H39. By referring to anatomical landmarks such as pulmonary vessels, fissures, and segmental bronchi, the different pulmonary lobes were identified on each CT section obtained at baseline and H39 and manually delineated using the roller ball of the computer. As the CT scan at H39 in the surfactant group was performed within three hours following the third bolus of HL-10, the increase of volume of tissue at H39 provided an estimated volume of the third bolus of HL-10. Therefore, the increase in volume of tissue at H39 was compared with the volume of HL-10 intratracheally administrated. The distribution of surfactant between upper and lower lobes was computed as the increase in lung tissue in each lobe.

### Statistical analysis

The normal distribution of data was verified by a Kolmogorov-Smirnov test. Patients' characteristics and regional changes in volumes of gas and tissue between day 7 and baseline were compared with a chi-squared test or an unpaired bilateral student test. Gas and tissue volumes at baseline and their changes between H39 and baseline within the lobes were compared by Friedman repeated measures analysis of variance on ranks followed by a Tukey test. Correlations between instilled volume of HL-10 and increase of tissue volume were made by linear regression. Cardiorespiratory and CT variables measured at different days were compared between the two groups using a two-way analysis of variance for a repeated factor and a grouping factor. The statistical analysis was performed with Sigmastat 3.1 (Systat Software Inc., Point Richmond, CA, USA). Data were expressed as mean ± standard deviation or median and interquartile range (25 to 75%) according to the data distribution. The statistical significance level was fixed at 0.05.

## Results

### Patients

Among the 20 patients, one in the surfactant group died at day 4 from severe hypoxemia. Of patients with ALI/ARDS, 30% were related to extrapulmonary sepsis. The overall mortality rate was 30%. As shown in Table 1, the clinical characteristics and cardiorespiratory parameters at baseline were not different between the control and surfactant patients.

**Table 1:** Baseline clinical characteristics of the patients

Variables	Control	Surfactant	*P* value
	(*n* = 10)	(*n* = 10)	
Male/female	9/1	8/2	NS
Age (years)	59 ± 16	62 ± 12	NS
SAPS II	40 ± 10	41 ± 10	NS
LISS	2.3 ± 0.4	2.6 ± 0.5	NS
Septic shock (%)	80%	70%	NS
Survival (%)	70%	70%	NS
Cause of ALI/ARDS			
*Bronchopneumonia*	4	6	
*Aspiration pneumonia*	1	1	NS
*Lung contusion*	2	0	
*Sepsis*	3	3	
PaCO_2 _(mmHg)	38.4 ± 8.2	37.9 ± 7	NS
PaO_2_/FiO_2 _(mmHg)	200 ± 63	201 ± 64	NS
TV/kg (ml)	5.7 ± 0.8	6 ± 0.9	NS
RR (breaths/min)	23 ± 4	20 ± 6	NS
Ppeak (cmH_2_O)	32 ± 5	32 ± 6	NS
Pplat (cmH_2_O)	23 ± 4	23 ± 5	NS
PEEP (cmH_2_O)	9.7 ± 0.9	9.4 ± 1	NS
Crs (ml.cmH_2_O^-1^)	38 ± 12	41 ± 23	NS
HR (beats/min)	110 ± 23	88 ± 24	NS
MAP (mmHg)	86 ± 15	87 ± 20	NS

ALI, acute lung injury; ARDS, acute respiratory distress syndrome; Crs, compliance of respiratory system; FiO_2_, fraction of inspired oxygen; HR, heart rate; LISS, lung injury severity score; MAP, mean arterial pressure; NS, not significant; PaCO_2_, partial pressure of arterial carbon dioxide; PaO_2_, partial pressure of arterial oxygen; Ppeak, peak airway pressure; PEEP, positive end-expiratory pressure; Pplat, plateau airway pressure; RR, respiratory rate; SAPS II, simplified acute physiology score II; Surfactant, porcine-derived lung surfactant; TV, tidal volume.

Data are expressed as mean ± standard deviation.

### Cardiorespiratory changes in control and surfactant groups

**Figure 2:**
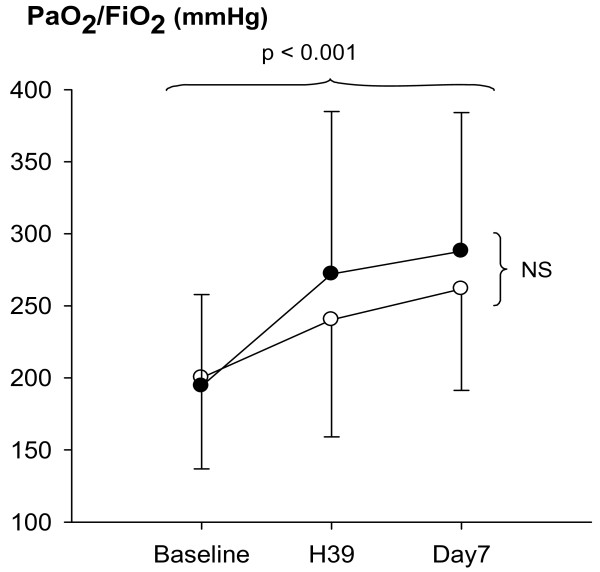
**PaO_2_/FiO_2 _ratio at baseline, 39 hours after baseline (H39) and day 7 in control (open circles) and surfactant groups (closed circles) of patients with acute lung injury/acute respiratory distress syndrome**. FiO_2_, fraction of inspired oxygen; PaO_2_, partial pressure of arterial oxygen.

As shown in Figure 2, PaO_2_/FiO_2 _ratio increased significantly from baseline to H39 and day 7 in both groups and in similar proportions. All other cardiorespiratory parameters remained unchanged between baseline and day 7 in both groups.

### Distribution of HL-10 in the lungs

**Figure 3:**
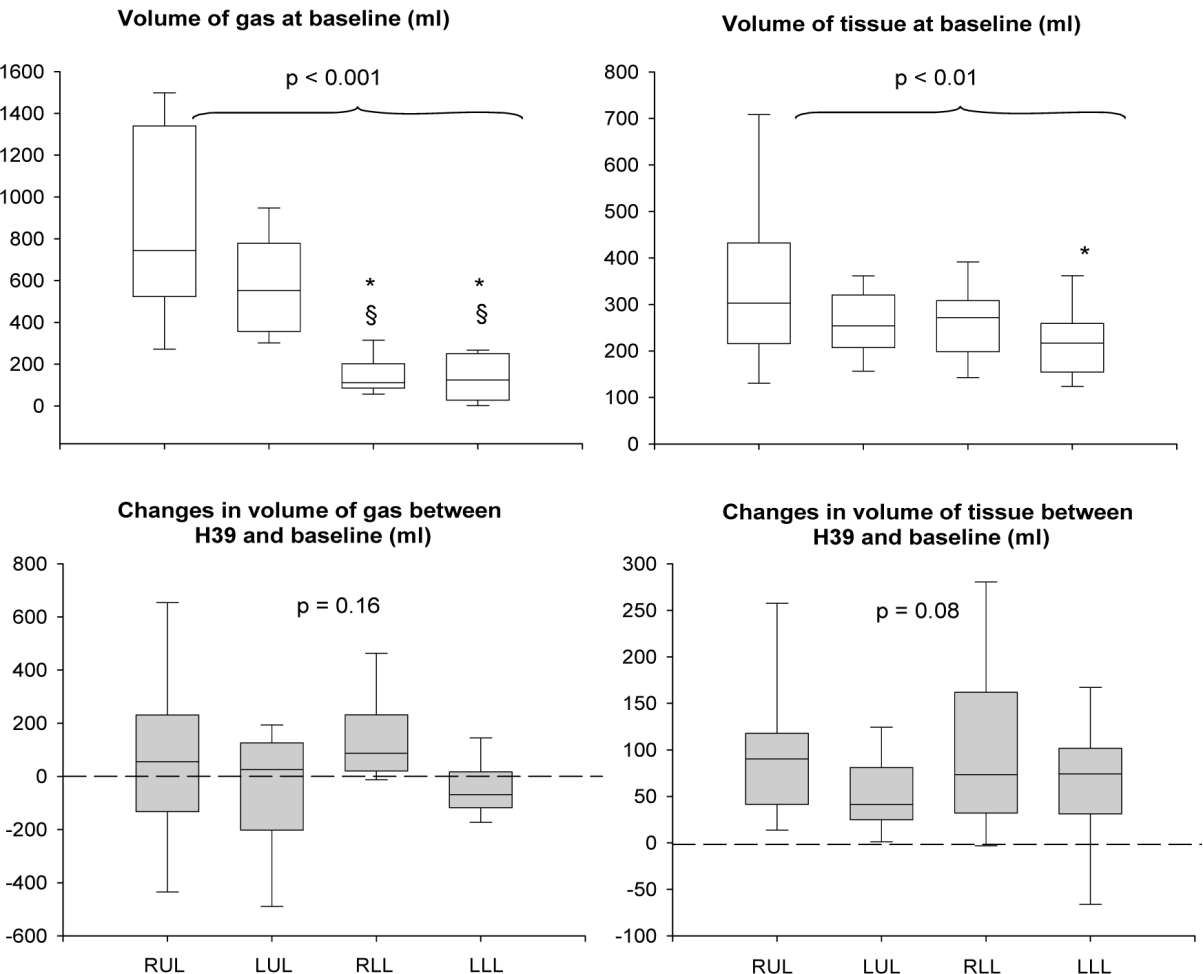
**Volumes of gas and tissue at baseline before HL-10 instillation (upper part of the figure) and changes in volume of gas and tissue between H39 (within three hours following the third bolus of HL-10) and baseline (lower part of the figure)**. Results shown in right upper and middle lobes (RUL), left upper lobe (LUL), right lower lobe (RLL) and left lower lobe (LLL) in patients with acute lung injury/acute respiratory distress syndrome instilled with 200 mg/kg of HL-10. Comparisons were performed by Friedman repeated measures analysis of variance on ranks followed by a Tukey test. *P *values above the horizontal brackets indicate significant difference between RUL, LUL, RLL and LLL using Friedman repeated measures analysis of variance.* *P *< 0.05 *versus *RUL, ^§ ^*P *< 0.05 *versus *LUL.

The mean volume of HL-10 instilled into the lungs per instillation was 240 ± 30 ml. In the surfactant group, between H39 (immediately after the third administration of HL-10) and baseline, CT tissue volume increased by 311 ± 200 ml. The increase in tissue volume correlated linearly with the instilled volume (R = 0.81, *P *= 0.008, Y = -987 + 5.4X). As shown in Figure 3, at baseline, CT gas volume was significantly less in lower lobes than in upper lobes whereas tissue volume was significantly greater in the right upper lobe than in left lower lobe. At H39, gas volume remained unchanged whereas tissue volume significantly increased in similar proportion in the upper and lower lobes.

In the control group, gas volume was not different between baseline and H39. Tissue volume of right lower lobe decreased significantly at H39 compared with the value of baseline (Table 2).

**Table 2:** Volumes of gas and tissue at baseline and H39 in the control group of patients

	Baseline	H39	*P* value
**Volume of gas (ml)**			
Right upper and middle lobe	864 ± 440	934 ± 411	NS
Left upper lobe	752 ± 321	722 ± 295	NS
Right lower lobe	178 ± 206	241 ± 244	NS
Left lower lobe	142 (47-277)	111(23-296)	NS
**Volume of tissue (ml)**			
Right upper and middle lobe	317 ± 115	313 ± 105	NS
Left upper lobe	281 ± 69	272 ± 71	NS
Right lower lobe	321 ± 106	275 ± 87	0.02
Left lower lobe	299 ± 89	267 ± 49	NS

Data are expressed as mean ± standard deviation or median and 25 to 75% interquartile range. H39, CT scan performed 39 hours after baseline. NS, not significant.

### Assessment of lung reaeration after HL-10 replacement

**Figure 4:**
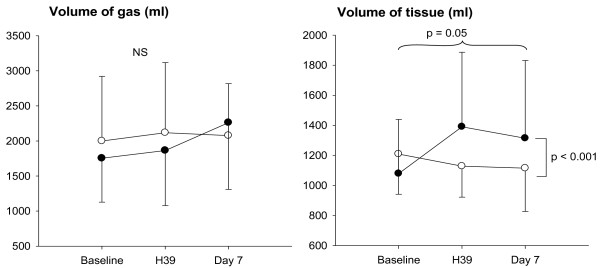
**Computerized tomography assessment of total gas and tissue volumes at baseline, 39 hours after baseline (H39) and day 7, in control (open circles) and surfactant groups of patients (closed circles)**.

At baseline and PEEP 10 cmH_2_O, total lung volume, gas volume and tissue volume were not different between control and surfactant groups. As shown in Figure 4, total gas volume did not change significantly between baseline, H39 and day 7 in control and surfactant groups. In contrast, HL-10 induced a significant increase in tissue volume at H39 that persisted at day 7 (interaction *P *< 0.001). The increase in tissue volume between day 7 and baseline correlated linearly with the instilled volume of HL-10 (R = 0.72, *P *= 0.03, Y = -1594 + 7.6X). Hyperinflated lung volume was not different between both groups at baseline, H39 and day 7.

**Figure 5:**
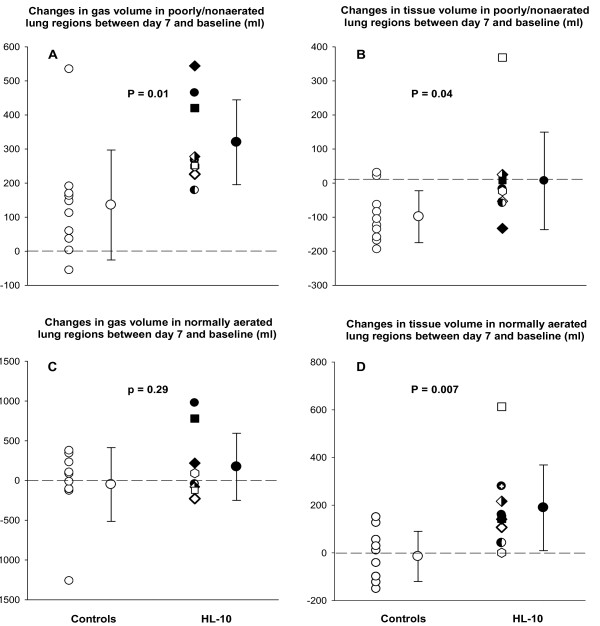
**Individual and mean changes in volume of gas and tissue in poorly/nonaerated lung regions (upper part of the figure) and normally aerated lung regions (lower part of the figure)**. Volume changes were measured on computed tomography scans acquired at baseline and seven days in patients who received either usual care (control, open circles) or usual care plus intratracheal porcine-derived surfactant (HL-10, closed circles). In the surfactant group, each patient is identified by a specific symbol.

As shown in Figure 5, in poorly or nonaerated lung regions, gas volume significantly increased at day 7 compared with baseline in both control and surfactant groups. The increase in gas volume at day 7 was significantly greater in the surfactant group than in the control group (320 ± 125 ml versus 135 ± 161 ml, *P *= 0.01, Figure 5a). In the control patients, tissue volume of poorly or nonaerated lung regions significantly decreased (Figure 5b, *P *= 0.04) between day 7 and baseline whereas it remained unchanged in surfactant group.

In normally aerated lung regions, gas volume did not change between day 7 and baseline in both groups (Figure 5c). However, HL-10 induced a significant increase in tissue volume at day 7 (189 ± 179 ml versus -15 ± 105 ml, *P *= 0.007, Figure 5d).

## Discussion

The present study demonstrates that intratracheal administration of porcine-derived surfactant to patients with ALI/ARDS induces a significant lung reaeration of poorly or nonaerated lung regions. This beneficial effect, however, is associated with a significant increase in lung tissue in normally aerated lung areas at day 7 whose mechanisms remain to be elucidated.

Distribution of surfactant within the lung is likely to be an important factor that determines the efficacy of surfactant therapy. Delivery technique and lung morphology influence surfactant distribution. In a previous randomized clinical trial [7], the unsuccessful surfactant treatment was related to the technique of aerosolization that provided less than 10% distal lung deposition [21]. Intratracheal instillation by a catheter positioned just above the carina has been shown to be much more effective in animals and patients with ARDS [6,22]. In patients with ARDS/ALI, the loss of lung aeration does not have a uniform distribution and, in the supine position, dependent and caudal lung regions are virtually nonaerated as a result of external compression by the abdomen and heart [13,14]. The distribution of exogenous surfactant in aerated and nonaerated parts of the distal lung has never been assessed and it is unknown whether instilled surfactant does penetrate into nonaerated lower lobes. In the present study, the CT scan at H39 in the surfactant group was performed within three hours following the third administration of HL-10. Based on the fact that the tissue volume did not change at H39 compared with its baseline value in the control group, we can assume that the increase in lung tissue between baseline and H39 in the surfactant group is representative of instilled exogenous surfactant. The present data show that the overall volume of instilled HL-10 was homogeneously distributed between upper and lower lobes and between normally and poorly or nonaerated lung regions (Figure 3). This result demonstrates that the procedure of instillation (successive bolus in right and left lateral positions followed by consecutive recruitment maneuvers) resulted in uniform bilateral surfactant distribution. A predominant distribution of HL-10 in normally aerated lung regions can be ruled out.

Although several randomized trials have failed to demonstrated beneficial effects of exogenous surfactant in adults patients with ARDS in terms of mortality and ventilator-free days [7,8,23], the effect of surfactant therapy on lung aeration had never been evaluated. In the present study, using CT regional analysis of normally and poorly or non-aerated lung regions, a significant higher lung reaeration was evidenced at day 7 in patients treated by surfactant replacement as compared with control patients (Figure 5a). This finding provides evidence that tracheal instillation of HL-10 induces a substantial and prolonged reaeration of poorly or nonaerated lung regions and more specifically of nonaerated lower lobes. This encouraging result supports the rationale for exogenous surfactant replacement as indication for lung reaeration in adult patients with ALI/ARDS.

HL-10-induced lung reaeration was, however, associated with a long lasting increase in lung tissue in previously normally aerated lung areas. Its mechanism remains unknown and several hypotheses can be discussed. A delayed alveolar clearance of the large doses of HL-10 administered to aerated lung regions, where endogenous surfactant is already present, is a possible mechanism that could explain the sustained increase in lung tissue. In newborn infants, the surfactant half life is around 35 hours [24]. In patients with ARDS treated by recombinant surfactant, components of exogenous surfactant were retrieved in bronchoalveolar lavage (BAL) two days after initial administration, but were no longer detectable five days later [6]. The dose of surfactant used in the present study was orders of magnitude beyond what was commonly used in neonates, older children and adults. The high volume of phospholipids administered may have prolonged the turn-over time, explaining the persistent increase in lung tissue. Another hypothesis explaining the increase of lung tissue could be an inflammatory reaction resulting from the interaction of HL-10 with active endogenous surfactant present in aerated lung regions [25]. As illustrated in the present study, normally aerated lung regions in ARDS/ALI are characterized by an excess of lung tissue [15] and an increased vascular permeability [26], two abnormalities increasing the vulnerability of lung parenchyma to external aggressions. In these regions, saline diluted HL-10 could induce depletion of endogenous surfactant [27], increased release of TNF and IL-6 in response to overinflation [28] and a resulting increase in lung micovascular permeability. The consecutive influx of albumin into the alveolar space could inactivate further endogenous surfactant [29], and aggravate lung injury. In addition, 720 ml of saline (4 ml/kg/bolus) containing HL-10 were instilled in both lungs over 36 hours. By itself, such an amount of liquid could induce lung injury in experimental normal lungs. Lastly, breakdown products of the phospholipids in surfactant, specifically lysophosphatidylcholine, can provoke inflammation. In this study, BAL after surfactant replacement was not performed. Further study is required to explore the correlation between the presence of inflammatory mediators, components of exogenous surfactant, protein and cells in BAL, and the CT increase in lung tissue in normally aerated lung areas.

Exogneous surfactant has strong immunomodulatory properties [30-32]. In patients with ARDS, exogenous surfactant therapy decreases IL-6 concentrations in the plasma and BAL of patients with ARDS, suggesting either a direct anti-inflammatory effect or a reduction of ventilator-induced lung stretch [6]. However, in the present study, despite surfactant-induced recruitment of poorly or nonaerated lung regions, CT lung hyperinflation was similar in both groups. Unexpectedly, HL-10-induced reaeration was not associated with a significant improvement in arterial oxygenation. Very likely, HL-10 instillation in normally aerated lung regions worsened regional ventilation/perfusion ratios through an increase in lung tissue. In other words, benefit in terms of aeration of poorly or nonaerated regions of the lung was likely to be counteracted by a negative impact of HL-10 on aeration of previously normally aerated lung.

## Conclusions

Although the rationale for exogenous surfactant replacement in patients with ARDS/ALI is strong with some phase II studies showing positive responses [33,34], all clinical phase III studies failed to demonstrate a beneficial effect in terms of mortality and duration of ventilation [7,8,12]. Our study demonstrates that non-selective tracheal administration of porcine-derived surfactant reaerates poorly or nonaerated lung regions, but induces a prolonged increase in lung tissue in regions remaining normally aerated; therefore, gas exchange is not improved. Further studies are needed to examine whether a more selective instillation of exogenous surfactant in poorly or nonaerated lung regions would be beneficial in terms of improvement of oxygenation, reduction of mortality and ventilator-free days.

## Key messages

• Intratracheal surfactant replacement reaerates pooly and nonaerated lung regions in patients with ALI/ARDS.

• Intratracheal surfactant replacement induces a prolonged increase in lung tissue in normally aerated lung regions.

## Abbreviations

ARDS: acute respiratory distress syndrome; ALI: acute lung injury; BAL: bronchoalveolar lavage; CT: computed tomography; FiO2: fraction of inspired oxygen; IL: interleukin; PaO2: partial pressure of arterial oxygen; PBW: predicted body weight; PEEP: positive end-expiratory pressure; TNF: tumor necrosis factor.

## Competing interests

The authors declare that they have no competing interests.

## Authors' contributions

QL carried out the study and drafted the manuscript. MZ, CG, and BB participated in the study and study analysis. JK participated in the interpretation of the results and gave the advices for improving the manuscript. JJR initiated the study, participated in the design of the protocol and helped to draft the manuscript. All authors read and approved the final manuscript.

## Supplementary Material

Additional file 1 **Computed tomography measurement of lung volumes of gas and tissue**. The detail method of computed tomography measurement of volumes of gas and tissue is described. Click here for file
