# Supplementary material for: Computed tomography assessment of exogenous surfactant-induced lung reaeration in patients with acute lung injury
Source: Crit Care. 2010 Jul 15;14(4):R135. doi: 10.1186/cc9186 (PMC2945105; doi:10.1186/cc9186)
Supplement: Additional file 1 — Computed tomography measurement of lung volumes of gas and tissue. The detail method of computed tomography measurement of volumes of gas and tissue is described. [file cc9186-S1.DOC]

**Computed tomography assessment of exogenous surfactant-induced lung reaeration in patients with acute lung injury**

Qin Lu^1^, Mao Zhang^2^, Cassio Girardi^3^, Belaïd Bouhemad^1^, Jozef Kesecioglu^4^ and Jean-Jacques Rouby^1^

From the Multidisciplinary Intensive Care Unit, Department of Anesthesiology and Critical Care Medicine, Assistance Publique-Hôpitaux de Paris, La Pitié-Salpêtrière Hospital, UPMC Univ Paris 06, 47-83 boulevard de l’hôpital, 75013 Paris, France

**Additional file 1**

**Computed tomography measurement of lung volumes of gas and tissue**

Lung scanning was performed from the apex to the lung base using a fast spiral Tomoscan SR 7000 (Philips, Eindhoven, The Netherlands). All images were observed and photographed at a window width of 1,600 HU and a level of -700 HU. The exposures were taken at 120 kV and 250 mA. The value of the pitch was 1. Contiguous axial CT sections 5 mm thick were reconstructed from the volumetric data.

The lung volume was computed as the total number of voxels present in a given region of interest times the volume of the voxel. The lung is composed of gas and tissue. The volumes of gas and tissue were measured according to a previously described analysis based on the tight correlation existing between the CT attenuation and the physical density. The CT attenuation characterizing each individual voxel is defined as the attenuation coefficient of the radiography by the material being studied minus the attenuation coefficient of water divided by the attenuation coefficient of water and is expressed in Hounsfield units (HU). By convention, the CT attenuation of gas is -1000 HU and the CT attenuation of water is 0 HU. Because lung tissue has a physical density very close to water density, a lung area characterized by a mean CT number of -500 HU is considered as being composed of 50% gas and 50% tissue. A lung area characterized by a mean CT number of -200 HU is considered as being composed of 20% gas and 80% tissue. Using this analysis, it was possible to compute the volume of gas and tissue present in a given lung region of interest. In a first step, the distribution of CT numbers was measured on each CT section for 256 compartments between -1200 HU and +200 HU, each compartment corresponding to an interval of 5.47 HU. For each compartment of a known number of voxels, the total volume, the volume of gas and tissue, and the fraction of gas were computed using the following equations:

1. Volume of the voxel = (size of the pixel)^2^ x section thickness

2. Total lung volume = number of voxels x volume of the voxel

3. Volume of gas = (-CT number / 1000) x total volume, if the compartment considered has a CT number below 0 (volume of gas = 0 if the compartment considered has a CT number above 0)

4. Volume of lung tissue = (1 + CT number / 1000) x total volume, if the compartment considered has a CT number below zero

5. Volume of lung tissue = number of voxels x volume of the voxel, if the compartment considered has a CT number above zero

where CT number is the CT attenuation of the compartment analyzed.

In a second step, the volumes of gas and tissue of each region of interest were calculated by adding the values of all the compartments present within the region of interest considered. In a third step, the volumes of gas and tissue of the overall lung were calculated by adding the volumes of each region of interest of the lungs. The overall lung volume at end expiration was defined as lung volume (gas + tissue). A specifically designed software (Lungview, Institut National des Télécommunications, France) was used to calculate regional lung volumes with different degrees of aeration. The optical disk on which rough CT data had been recorded was downloaded in a PC equipped with Lungview, and each CT image was displayed on a 21-in. screen. The roller ball of the computer was used for manually delineating left and right lung parenchymas chosen as regions of interest.
